# A Chinese *Chan*-Based Mind-Body Intervention Improves Sleep on Patients with Depression: A Randomized Controlled Trial

**DOI:** 10.1100/2012/235206

**Published:** 2012-04-30

**Authors:** Agnes S. Chan, Queenie Y. Wong, Sophia L. Sze, Patrick P. K. Kwong, Yvonne M. Y. Han, Mei-chun Cheung

**Affiliations:** ^1^Department of Psychology, The Chinese University of Hong Kong, Shatin, New Territories, Hong Kong; ^2^Integrative Neuropsychological Rehabilitation Center, The Chinese University of Hong Kong, New Territories, Hong Kong; ^3^Henan Songshan Research Institute for Chanwuyi, Henan 452470, China; ^4^Division II, Kwai Chung Hospital, New Territories, Hong Kong; ^5^Department of Special Education and Counselling, The Hong Kong Institute of Education, Tai Po, Hong Kong; ^6^Institute of Textiles and Clothing, The Hong Kong Polytechnic University, Kowloon, Hong Kong

## Abstract

Sleep disturbance is a common problem associated with depression, and cognitive-behavioral therapy (CBT) is a more common behavioral intervention for sleep problems. The present study compares the effect of a newly developed Chinese *Chan*-based intervention, namely *Dejian* mind-body intervention (DMBI), with the CBT on improving sleep problems of patients with depression. Seventy-five participants diagnosed with major depressive disorder were randomly assigned to receive 10 weekly sessions of CBT or DMBI, or placed on a waitlist. Measurements included ratings by psychiatrists who were blinded to the experimental design, and a standardized questionnaire on sleep quantity and quality was obtained before and after the 10-week intervention. Results indicated that both the CBT and DMBI groups demonstrated significantly reduced sleep onset latency and wake time after sleep onset (effect size range = 0.46–1.0, *P* ≤ 0.05) as compared to nonsignificant changes in the waitlist group (*P* > 0.1). Furthermore, the DMBI group, but not the CBT or waitlist groups, demonstrated significantly reduced psychiatrist ratings on overall sleep problems (effect size = 1.0, *P* = 0.00) and improved total sleep time (effect size = 0.8, *P* = 0.05) after treatment. The present findings suggest that a Chinese *Chan*-based mind-body intervention has positive effects on improving sleep in individuals with depression.

## 1. Introduction

About 80% of individuals with major depressive disorder report sleep disturbances [1, 2]. Typical sleep problems in depressed patients can be manifested as difficulty in falling asleep at sleep onset (initiation insomnia) and/or frequent premature awakenings (maintenance insomnia). Subjective and objective sleep disturbances have been found to be associated with perpetuating mood disturbance and slowing the remission process of depression [3–5]. Sleep disturbances not only frequently coincide with depressive symptomatology, but are also one of the most common and persistent residual symptoms in remitted depression and precipitating factors for relapse and recurrence of depressive episodes [6–8]. Empirical findings have suggested that over 90% of individuals continue to report poor sleep quality after effective depression treatment [9, 10], and sleep disturbance acts as an independent predictor for depression recurrence in individuals with a prior history of depression [11]. Given the close connection between depression and sleep disturbances, effective treatment for comorbid or residual sleep abnormalities in full-blown or remitted depression would be important for alleviating current depressive symptoms and preventing relapses.

Currently, sleep disturbances in depressed patients are sometimes treated with pharmacotherapy, probably due to its instantaneous and efficacious effect. However, evidence for long-term efficacy (i.e., >4 weeks) of hypnotics is still unknown, and several problems (e.g., altered sleep stages and dependence) are likely to arise during or after pharmacotherapy [12–14]. Thus, it is clinically significant to explore psychological and behavioral interventions that may improve insomnia. However, studies on psychological and behavioral interventions for improving sleep quality in depression are relatively sparse [15–20].

Among the relatively limited empirical studies on psychological interventions for sleep problems in depressed patients, the cognitive-behavioral therapy (CBT) is comparatively well-established and well-studied. One study reported that after 6 sessions of CBT, patients with depression showed significant improvement in sleep, including shorter sleep onset latency (SOL) and wake time after sleep onset (WASO), and longer total sleep time (TST) [18]. Another study also reported that, depressed patients who received concurrent treatment with antidepressants and CBT showed higher rates of remission of depression and from insomnia, as compared with patients who received a quasidesensitization treatment [19]. Similar results were reported in that psychiatric patients who did not respond to medication for their chronic insomnia showed significant improvement after 6 sessions of behavioral treatment, and the effect was sustained after 6 to 12 months [15].

Since psychological intervention for sleep problems of patients with depression is a relatively less studied topic, the present study will examine the effectiveness of a newly developed Chinese* Chan*-based mind-body intervention as a possible treatment for sleep disturbance. This intervention, termed *Dejian* mind-body intervention (DMBI) [21], was developed upon the medical principle of the *Shaolin* Temple. The intervention consists of psychosocial education, mind-body exercises, and diet modification. There have been studies that revealed the positive effects of this intervention on reducing depressive mood in a group of community-dwelling sample, and on improving some aspects of their physical health [22]. Other studies have also suggested that this intervention is effective in improving cognitive functions and behavioral problems of patients with developmental and acquired brain disorders [23–25]. A recent electrophysiological study found that one of the mind-body exercises of this intervention, namely, the Passive *Dan Tian* Breathing, could induce a relaxed and calm state of mind that may have a positive effect on mood and sleep [26]. In addition, this intervention has been applied at our centre for several years to treat patients with various psychological problems and cognitive disorders, with about 85% of the clients showing different levels of improvement. Given the encouraging empirical findings and clinical observations, the primary goal of the present study is to examine the effect of the DMBI on the sleep of patients with depression. It is hypothesized that the DMBI, as compared with the CBT, would demonstrate similar if not better effects, for improving sleep of patients with depression, and both the DMBI and CBT groups will demonstrate significant improvements as compared with the waitlist control.

## 2. Methods

### 2.1. Participants

A total of 75 participants between the ages of 28 and 62 and diagnosed with major depressive disorder and reported sleep disturbances were recruited from the West Kowloon Psychiatric Centre. All participants were screened by medical doctors and assessed to meet the diagnostic and statistical manual of mental disorders (DSM-IV-TR) [27] criteria for major depressive disorder, as determined by the structural clinical interview for the DSM-IV (Chinese-bilingual SCID-I/P version) [28] conducted by a clinical psychologist. Their SCID-based diagnosis revealed different severity levels of depression, including mild-to-moderate depressive episodes, or in partial remission. Participants with at least one of the following sleep problems were included in the study: difficulty initiating sleep, midnight awakenings, difficulty in resuming sleep after awakening, total sleep time of less than 6.5 hours per night, or on medication for improving sleep quality. Participants with a history of head injury, seizure, stroke, other central nervous system disease, other comorbid psychiatric illness, or report of strong suicidal ideation were excluded.

The 75 participants were randomly and equally assigned to one of the three groups: CBT, DMBI, and waitlist control. Randomization of patients to treatment conditions was conducted by a medical professional who was blind to the experimental design. The patients were also blind to the potential benefits of the two techniques. All participants were prescribed antidepressants and/or hypnotic medication, and continuously followed up by the psychiatrists.

For the two treatment groups, participants with <70% attendance rate were excluded from the study, which resulted in 18 participants in the CBT group and 17 participants in the DMBI group. In the waitlist control group, 16 participants remained in the group after 9 withdrew from the study at postassessment.  Table 1 presents the demographic characteristics, severity level of depressive symptoms, and sleep parameters of each group at the baseline. The three groups were matched by age (*F*(2,48) = 0.27, *P* = 0.77), education level (*F*(2,48) = 1.72, *P* = 0.19), gender (**χ*^2^*(2) = 1.49, *P* = 0.48), severity of depressive disorder based on the SCID clinical diagnosis (**χ*^2^*(4) = 3.06, *P* = 0.55), the total score on the 17-item Hamilton Psychiatric Rating Scale for Depression (HRSD) [29] (*F*(2,48) = 0.07, *P* = 0.94), and the proportion-prescribed antidepressants (**χ*^2^*(6) = 6.03, *P* = 0.42) and hypnotic drug (**χ*^2^*(2) = 0.12, *P* = 0.94).

### 2.2. Ethics

The study was conducted in accordance with the Helsinki Declaration of the World Medical Association Assembly. The research protocol was approved by the Joint The Chinese University of Hong Kong-New Territories East Cluster (Joint CUHK-NTEC) Clinical Research Ethics Committee (CREC) and the Kowloon West Cluster (KWC) CREC of the Hospital Authority in Hong Kong SAR. Written informed consent was obtained from all participants.

### 2.3. Procedure

 Each participant was individually interviewed by a psychiatrist and a research assistant on his/her quality and quantity of sleep, and depressive mood with standardized questionnaires at the baseline and then after 10 weeks. The psychiatrist and the research assistant who administered the assessments were blind to the experimental design and group assignment of the study. After the baseline assessment, the two treatment groups underwent 10 weekly 90-minute training sessions either on CBT or DMBI, whereas the waitlist control group did not receive any psychological intervention.

### 2.4. Interventions

 The structure and format of the treatment groups were designed to parallel each other, in terms of duration and frequency of sessions, group size, didactic teaching and learning elements, in-session sharing and discussion, and weekly home assignments. Throughout the 10 training sessions, participants in the treatment groups learned the fundamental principles and techniques of the corresponding interventions. They were requested to regularly practice the techniques at home, particularly with some techniques that might help to enhance sleep quality to be carried out at bedtime. In order to ensure the quality of the practice in techniques for both groups, the respective trainers observed and monitored each participant during each training session to ensure that the participants had correctly mastered the techniques.

#### 2.4.1. Chinese Chan-Based Mind-Body Intervention

This newly developed intervention is established on a Chinese* Chan *tradition, namely, *Chanwuyi* (i.e., Zen, martial art and healing), from the *Shaolin* Temple [30]. The intervention was termed DMBI [21] as named after the Grand Master of *Chanwuyi*—*Shi Dejian* (a *Shaolin* monk).

The principle of DMBI is to alleviate psychological distress by understanding the root of problems in accordance with Buddhism philosophy and enhance mental and physical health by practicing some of the *Shaolin Qigong *exercises (i.e., mind-body exercises) and refining the diet to reduce the intake of food that will generate excessive internal heat [21, 30]. A major difference of this intervention from conventional psychological interventions is that it emphasizes integrative treatment on the mind and the body, which, on the one hand, changes the thought process, and, on the other hand, improves mental and physical health by practicing mind-body exercises and refining the diet.

 The 10-session DMBI was delivered to the depressed patients by a clinical psychologist who has over 10 years of clinical experience and is familiar with the DMBI model. There were four treatment components taught and practiced throughout the intervention. First, the participants learned to “listen to their body,” that is, to increase awareness of how unrealistic desires (e.g., greed), anger, and obsession (e.g., craving for something or somebody) affect their mental and physical health. For instance, how s/he feels when having an argument with others and craving for something beyond reality. The participants were advised to be aware of some physical signs, such as headaches, shortness of breath, lack of appetite, stomachaches, constipation, or diarrhea.

Second, they were told about how some foods if taken, according to the *Shaolin* medical principle, would generate excessive internal heat, which would, in turn, adversely affect their mood and physical health. These foods include ginger, garlic, green onion, spicy foods, eggs, meat, and fish. Suggestions were made to the participants to reduce intake of such foods. It should be stressed that participants were not required to abstain from these foods but advised to cut down on their intake according to their own lifestyles and plans. The log record revealed that 54% of participants have reduced intake of all those specified foods, and 85% have reduced or abstained from consumption of eggs, ginger, garlic, green onion, and spicy foods. There was no specific advice on restricting the use of caffeinated drinks. In the meantime, participants were advised to consume food that are good for their health, including fresh vegetables (e.g., broccoli and tomatoes), fruits (e.g., grapes and apples), grain (e.g., noodles and brown rice), beans (e.g., soy and peas), mushrooms (e.g., black fungus and straw mushrooms), nuts (e.g., walnuts and almonds), and root vegetables (e.g., yams and taros). The participants were encouraged to take food from the above seven categories everyday with one to three kinds from each category. The type and amount of food were not specified, as long as they were fresh and seasonal, and the participants ate until they were 80% full for each meal. They were advised to gradually change their diet, and given guidance to feel the changes after modifying their diets. The progress of each subject was monitored by the psychologist during the group sessions.

Third, the participants learned to foster self-awareness and self-control of their mental state (i.e., keeping calm and relaxed when feeling distressed and angry) by practicing self-guided massages (i.e., *Qigong*), such as rolling their hands slowly up and down between the chest and the abdomen, resting their hands on their abdomen while quietly observing their breathing, and massaging their nasal bridge.

Fourth, the participants learned some *Shaolin* mind-body exercises that are beneficial to their mental and physical health. The exercises, somewhat like *Tai Chi*, are sets of slow movements that emphasize smooth, gentle, and calm movements. The principles and practicing details of a few basic *Shaolin* mind-body exercises were elaborated in the two published books [21, 30]. The functions of the exercises are to reduce stress, increase flexibility of the four limbs, enhance strength of the legs, and improve overall physical health and the circulation of *Qi* and blood. The practicing time was not fixed as participants were instructed to practice the exercises until they felt warm and relaxed, but not to the point of overexertion. The rationale for being flexible in practice is to facilitate participants to increase their sense of self-control and self-awareness (e.g., stop doing the exercise when feeling warm). The philosophy behind the *Shaolin* mind-body exercises is different from that of Western cardiovascular exercises, where the prior does not require oversweating, as a little warm feeling with a little sweating is sufficient to achieve blood and *Qi* circulation. According to the daily log record and weekly in-session monitoring, participants who had ≥70% attendance rate were able to comply with at least three of the four treatment components and reported regular practice of the treatment methods in their daily life.

#### 2.4.2. Cognitive-Behavioral Therapy (CBT)

The 10-session CBT protocol adopted in the present study was derived from a combination of sources, including *cognitive therapy for depression* by Beck and colleagues [31], *mind over mood* by Greenberger and Padesky [32], and *cognitive-behavioural therapy in groups* by Bieling, McCabe and Antony [33]. Typical treatment components included, (a) behavioral activation, (b) self-monitoring, (c) cognitive restructuring, including evaluating and reconsidering interpretive and predictive cognitions, (d) cognitive techniques for deeply held core beliefs and deep-rooted conditional assumptions, (e) rehearsal of coping skills, and (f) relapse prevention. Specific training on progressive muscle relaxation technique was provided and recommended for practice at bedtime every night to improve sleep. The participants were advised to follow a CD which provided guidance on a standard procedure that lasted about 20 minutes for each practice session.

The 10-session CBT was delivered by a clinical psychologist who has over 10 years of clinical experience and frequently applies this technique in her therapy sessions for hospital patients. At the beginning of the intervention, the participants received psychoeducation on the biopsychosocial model of depression and familiarized themselves with the relationship between mood state, cognition, and behavior. In each of the subsequent sessions, the therapist started with a review of the experiences of each participant over the past week and monitored the progress of each participant in their practice of the treatment techniques. Finally, the therapist would set the stage for homework near the end of the session.

### 2.5. Sleep Measures

The overall sleep problems of participants at pre- and post-intervention were evaluated by three psychiatrists who were blinded to the rationale and experimental design of the study. The evaluations were based on ratings by the psychiatrists, which used a three-point scale from “0” to “2” to measure three items: difficulties in falling asleep, difficulties in maintaining sleep during the night, and difficulties in resuming sleep after awakening, per the HRSD [29]. A higher score indicates greater difficulty and “0” indicates “no difficulty.” The sum of the three scores was computed for subsequent pre-post comparisons.

In addition to the psychiatrist rating, each participant was asked to report on his/her sleep problems in terms of TST, SOL, and WASO at pre- and post-intervention. The TST was measured as the total sleeping hours/night. The SOL was measured as minutes taken to fall asleep at sleep onset each night. The WASO was measured as minutes taken to resume sleep after a midnight awakening. The psychiatrist ratings of HRSD-sleep items (*F*(2,46) = 0.92, *P* = 0.41), TST of participants (*F*(2,47) = 0.38, *P* = 0.69), SOL (*F*(2,45) = 0.84, *P* = 0.44), and WASO (*F*(2,35) = 0.11, *P* = 0.90) were not significantly different among the groups at the baseline (Table 1).

Pre-post rating on overall level of energy on a four-point scale, from “0” to “3,” was also obtained, with a higher score indicating a greater extent of energy loss resulting in a negative impact on daily functioning. Finally, participants of the two treatment groups were asked to rate the treatment effect on their overall sleep quality, on a 7-point scale from “largely deteriorated” (−3) to “largely improved” (+3) with 0 indicating “no change” mainly after intervention. The above questions were selected because of their simplicity and concreteness.

### 2.6. Statistical Analysis

The SPSS (Version 15.0) statistical software package was used for data analysis. Baseline group differences in demographic and clinical characteristics and severity of mood and sleep problems were analyzed with one-way analysis of variance (ANOVAs) and chi-square tests. The main analyses investigating the treatment effects were performed by comparing pre- and post-intervention mean difference with a 95% confidence interval (CI) on various sleep measures using paired samples *t-*tests with planned comparisons for each group. Given that several participants had reported no difficulty on specific sleep measures at the baseline, they were excluded from the analyses, and thus the sample size of each group would vary across analyses. The pre-post changes in sleep measures were compared within and between groups using one sample *t*-tests and independent samples *t*-tests, respectively, with planned comparisons. Further subgroup analyses were performed using *t*-tests on participants who demonstrated more severe sleep problems, including that TST was less than 6 hours/night, SOL was more than 30 minutes/night, and WASO was more than 15 minutes/time at baseline. Effect size (Cohen's *d*) was also calculated both within and between group for each primary outcome measures.

## 3. Results

### 3.1. Overall Sleep Problems

The overall sleep problems of participants were evaluated by three psychiatrists who were blinded to the experimental design. At the baseline, the three groups of participants were matched on severity level of overall sleep problems as measured by the summation scores of the HRSD-sleep items (*P* = 0.41). After treatment, participants in both the DMBI and CBT groups showed some improvement as suggested by their reduced scores from pre- to post-treatment (−1.50 and −0.53 for the DMBI and CBT groups, respectively, Table 2). The extent of reduction in score for the DMBI group was significant (*t* = 3.99, *P* < 0.001, 95% CI = 0.70 to 2.30), while that of the CBT group was not (*t* = 1.04, *P* = 0.16, 95% CI = −0.58 to 1.64). In contrast, patients in the waitlist control group showed an increased score of 0.94 after 10 weeks which suggested an increased severity of sleep problems although the change was not statistically significant (*t* = −1.64, *P* = 0.06, 95% CI = −2.16 to 0.28). These results suggested that both CBT and DMBI had some effects on improving the sleep problems of patients with depression, but the effect of DMBI seemed to be more prominent.

 The psychiatrist ratings were consistent with the self-rating of the participants. That is, on a rating scale from “+3” to “−3,” the participants were asked if their overall sleep quality has improved or declined after the intervention (where “+3” represents “largely improved” and “+1” represents “somewhat improved”). The mean rating of the DMBI group was 1.76 (±0.90) which suggested the participants considered that DMBI had a positive effect on sleep quality improvement. The CBT group also indicated that the CBT techniques helped to improve their sleep (mean rating = 1.00 ± 1.12). In addition, the mean rating of the DMBI group was significantly higher than that of the CBT group (*t*(32) = 2.19, *P* = 0.04, 95% CI = 0.05 to 1.47) which suggested that the participants considered the DMBI more effective than the CBT for improving sleep. 

### 3.2. Quantitative Analyses on Improvement of Sleep

While the psychiatrist and participant ratings demonstrated that both treatment groups showed improvement in sleep with the DMBI group demonstrating a greater extent of improvement, further analyses were attempted to quantify the improvement in each dimension of sleep (including TST, SOL, and WASO) in more detail. 

#### 3.2.1. Total Sleep Time (Table 3)

At the baseline, the mean total hours of sleep per night were comparable among the three groups (*P* = 0.69). After the treatment, the DMBI group showed a significant increase in TST (mean increment = 47.64 minutes, *t *= −2.00, *P* = 0.03, medium effect size of 0.48, 95% CI = −98.4 to 3.0), while the CBT group did not show a significant improvement (mean reduction = 1.74 minute, *t* = 0.09, *P* = 0.47, 95% CI = −41.4 to 45.0), and the waitlist group showed a tendency of reducing TST (mean reduction = 40.02 minutes, *t* = 1.34, *P* = 0.10, 95% CI = −24.0 to 104.4). Since these results might be biased as they include individuals with less severe sleep problems, further analyses were conducted on individuals who had reported a TST of less than 6 hours per night. With this criterion, there were 7, 5, and 6 subjects in the waitlist, CBT and DMBI groups, respectively. As shown in Table 3, the waitlist (*t*(6) = −0.49, *P* = 0.32, effect size = 0.19, 95% CI = −76.8 to 51.0) and CBT (*t*(4) = 0.65, *P* = 0.23, effect size = 0.29, 95% CI = −59.4 to 154.8) groups showed no significant changes in the duration of sleep. In contrast, the participants in the DMBI group showed, on average, a one-hour sleep increment. Although the increment of the DMBI group was statistically nonsignificant (*t*(5) = −1.96, *P* = 0.05, 95% CI = −154.8 to 20.4), the effect size was large (0.80). Thus, the nonsignificant difference may be due to the small sample size (a minimum of 12 participants are required to reach statistical significance). 

#### 3.2.2. Sleep Onset Latency (Table 4)

At the baseline, the SOL among the groups was comparable (*P* = 0.44). After treatment, the DMBI group showed a significant reduction in SOL (mean reduction = 9.81 minutes, *t = *1.85, *P* = 0.04, medium effect size of 0.46, 95% CI = −1.51 to 21.14, Table 4). In contrast, the CBT group did not show much change (mean increment = 2.13 minutes, *t* = −0.41, *P* = 0.34, 95% CI = −13.17 to 8.92), and the waitlist control group showed a tendency of increased latency of sleep (30% increment, mean increment = 13.5 minutes, *t* = −0.87, *P* = 0.20, 95% CI = −46.70 to 19.70). In addition, further analyses were conducted on subgroups who had greater difficulties in falling asleep (i.e., requiring more than half an hour to fall asleep) at the baseline. There were 4, 5, and 6 subjects in the waitlist, CBT and DMBI groups, respectively. As shown in Table 4, both the CBT (mean reduction = 13 minutes, 95% CI = −3.19 to 29.19) and DMBI (mean reduction = 18 minutes, 95% CI = −14.63 to 49.63) groups showed a reduction in SOL. Although the reduction did not reach statistical significance, the effect size for the CBT group was 1.00 which was large, and that of DMBI group was 0.57, which was medium. In contrast, the waitlist group showed an increase in SOL (mean increment = 23 minutes, 95% CI = −68.20 to 23.20), and the effect size was large (0.78). Thus, these results suggested that both CBT and DMBI had positive effects on helping patients with depression to fall asleep faster, with the effect of the DMBI being more prominent in some aspects. 

#### 3.2.3. Wake Time after Sleep Onset (Table 5)

At the baseline, the mean time needed to resume sleep after awakening was about 30 minutes for the three groups, and the difference was not significant (*P* = 0.90). After treatment, the reduction of time to resume sleep in the waitlist group was 0.55 minute (95% CI = −23.46 to 24.56), 7.97 minutes (95% CI = −3.99 to 19.93) for the CBT group, and 12.10 minutes (95% CI = −6.74 to 30.94) for the DMBI group (Table 5). The DMBI group showed a greater amount of reduction and its effect size approached a medium degree (0.46). The data were further analyzed on a sub-group who had greater problems in this aspect (i.e., taking longer than 15 minutes to resume sleep after awakening) at the baseline. As a result, there were 5, 9, and 6 subjects in the waitlist, CBT and DMBI groups, respectively. As shown in Table 5, the DMBI and CBT groups showed a mean reduction of 23 and 21 minutes, respectively. The reduction in the CBT group was significant (*t*(8) = 2.98, *P* = 0.01, 95% CI = 4.66 to 36.45), and that in the DMBI group approached significance (*t*(5) = 1.96, *P* = 0.05, 95% CI = −7.11 to 52.78), with a large effect size for both groups (DMBI = 0.80; CBT = 0.99). In contrast, the waitlist group did not show much change in time taken to resume sleep (pre-post reduction = 5.5 minutes, 95% CI = −54.87 to 65.87). Again, these data seemed to be consistent with the above analyses, which suggested that both the CBT and DMBI had positive effects on sleep, with those who practiced DMBI showing greater improvement. 

### 3.3. Mood and Level of Energy

Given the close connection between insomnia and depressive mood, the three groups were also compared on the degree of changes in their HRSD total scores rated by the psychiatrists. Both treatment groups showed a 43% to 46% improvement in HRSD scores (DMBI: from 12.06 ± 4.48 to 6.88 ± 4.47; CBT: from 11.94 ± 3.98 to 6.44 ± 5.78). Paired sample *t* statistics revealed that both treatment groups were rated to have significantly reduced severity of depressive symptoms after intervention (DMBI: *t*(16) = 3.85, *P* = 0.001, 95% CI = 2.32 to 8.03; CBT: *t*(17) = 4.55, *P* = 0.000, 95% CI = 2.95 to 8.05), whereas there was no significant improvement (mean scores changed from 11.50 to 10.19) observed in the waitlist control group (*t*(15) = 0.82, *P* = 0.21, 95% CI = −2.11 to 4.74). Consistent with the results above, both CBT and DMBI have positive effects on improving the mood of patients with depression, which, in turn, may be related to improvement in their sleep. 

Given that improvement in sleep is also associated with higher energy level during daytime, it is anticipated that CBT and DMBI which have demonstrated to contribute to improvement in sleep should also show improvement for daytime energy level. A pre-post comparison was performed through self-rating (from 0 to 3) of energy loss during daytime for each group of participants. It was found that both the CBT and DMBI groups reported significantly less problem of reduced energy level during the daytime after treatment (CBT: mean reduction of 0.53 point, *t*(16) = 3.50, *P* = 0.002, effect size = 0.85, 95% CI = 0.21 to 0.85; DMBI: mean reduction of 0.62 point, *t*(16) = 2.86, *P* = 0.006, effect size = 0.70, 95% CI = 0.17 to 1.13). In contrast, the waitlist group did not show any significant changes (*t*(15) = 1.47, *P* = 0.08, 95% CI = −0.12 to 0.66). 

### 3.4. Association between Sleep Improvement and Education Level

To explore a possible factor that might explain the different extent of improvement in the CBT and DMBI groups, the level of education within the groups was further analyzed. In our clinical experience, individuals with lower level of education have more difficulty in mastering the relatively more complicated CBT technique; it is one of the rationales for the development of the DMBI, which is a simpler method that can be easily followed by individuals with lower level of education and/or limited intelligence. The level of education in each group was correlated with the change in scores in the HRSD-sleep items. The results demonstrated that the DMBI group showed a significant negative correlation (*r* = −0.51, *P* = 0.045), which suggested that individuals with lower level of education showed greater extent of improvement. The CBT (*r* = −0.18, *P* = 0.52) and waitlist (*r* = 0.01, *P* = 0.97) groups did not show a significant correlation. This result is consistent with the hypothesis that the DMBI may be more acceptable for individuals with lower levels of education.

## 4. Discussion

The present study showed that both the CBT and DMBI had positive effects on improving sleep problems of patients with depression. Specifically, individuals with more severe sleep problems who had practiced CBT showed on average, a 13-minute reduction in SOL and 21-minute reduction in WASO, but no significant increase in TST. These findings are consistent with previous studies in Western countries which reported the effect of CBT on improving the initial and middle insomnia of patients with depression [15, 18, 19]. Among the few Western studies on CBT or CBT combined with antidepressants for depression comorbid with insomnia, there was about a 40–80% improvement in SOL and WASO. However, the extent of improvement in the present study (25–39%), although statistically significant, is less prominent than those studies. In addition to sleep improvement, the present study also found concurrent improvement in depressive mood as rated by the psychiatrists, which is in line with those reported in previous studies [17–19]. Thus, the present study suggests that CBT may be applicable to the Chinese population although the effect may be less prominent. 

Moreover, a major goal of the present study is to examine the effect of a relatively new intervention approach, namely, the DMBI. This Chinese *Chan-*based mind-body intervention, which originated from the *Shaolin* Temple in China and has been used for over a thousand years, has not been empirically studied until recent years. In the present study, patients with depression who have severe sleep problems demonstrated a one-hour increment in TST, 18-minute reduction in SOL, and 23-minute reduction in time to resume sleep after the intervention. They also showed significant improvement in overall sleep problems based on psychiatrist ratings. Similar to the CBT method, DMBI also demonstrated a concurrent positive effect on reducing depressive symptoms. While previous studies demonstrated the positive effects of DMBI on improving depressive mood and physical health problems of a community sample [22], the present results further suggest that this method can be applicable to the clinical population. 

As compared with the CBT group, the DMBI group in general demonstrated a greater extent of improvement in sleep. Specifically, individuals in the DMBI group were rated by the psychiatrists with significant improvement in overall sleep problems, which was not observed in the CBT group. In addition, the DMBI group demonstrated an average increment of one hour of sleep, while the CBT did not show significant improvement in the TST. Furthermore, individuals in the DMBI group showed a reduction of 18 minutes in SOL and 23 minutes in WASO, while those in the CBT group showed a 13-minute reduction in SOL and 21-minute reduction in WASO. 

There are two possible reasons for the more prominent effect of DMBI as compared with the CBT in the present study. First, the participants in the present study have a lower mean education level (about 9 years) than those in the western studies (≥12 years). From our clinical experiences, some individuals with lower level of education or limited intelligence have difficulties in following the instructions of the CBT. On the other hand, the dietary recommendation and mind-body exercises of the DMBI are relatively concrete and simple to understand and master, even for less educated or less intelligent people. We have clinically applied the DMBI on individuals with dementia or mental retardation which has yielded positive results [24, 25]. In addition, the present finding of significant correlation between education level and degree of improvement in sleep measure within the DMBI group also provides some insight for this hypothesis. Thus, lower level of education of the subjects may be a factor that affects the present results. In addition, the present study suggests that the DMBI may be more suitable for populations with special needs, such as patients with Alzheimer's disease or autism who cannot follow the instruction of the CBT. 

Second, according to the principle of the DMBI model, there are no strict regulations on diet modification and duration of practicing mind-body exercises. The participants were only recommended to reduce the intake of certain foods according to their own lifestyles and plans, and practice the exercises in response to their bodily signals. This sort of “doing it naturally” approach may be more well accepted by patients with depression who are usually less motivated in tasks that require much effort, as compared to some techniques of the CBT, such as the progressive muscle relaxation which requires about 20–30 minutes to practice each time. Therefore, the DMBI may be particularly suitable for individuals who cannot comply with the CBT method. 

Given the encouraging results, the DMBI may have potential as a complementary intervention for treating clinical populations with complaints of sleep disturbances given its cost effectiveness and easiness to comprehend and practice. Despite the encouraging findings, the present study has several limitations. The present study has provided evidence for the short-term effect of a 10-week DMBI training, yet its long-term effect remains unknown, which warrants further study. Furthermore, given that the results of the subgroup analyses on individuals with more severe sleep problems had a moderate-to-large effect size, but did not reach statistical significance, future studies with a larger sample size are needed to further validate the effects of DMBI. Another weakness is that an intention to treat analysis is not adopted in present study, which may be attempted in future study to further verify the effects of DMBI. Finally, the present study has adopted subjective rating questionnaires; hence, more objective polysomnographic sleep measures and event-related sleep electroencephalographic measures may be adopted in future studies to further validate the results. 

## Figures and Tables

**Table 1:** Demographic and clinical characteristics at baseline.

Characteristics	WL (*n* = 16)	CBT (*n* = 18)	DMBI (*n* = 17)	*P*
Mean age (years)	45.44 (8.25)	47.39 (6.63)	47.06 (9.54)	0.77
Mean education (years)	8.06 (2.59)	9.78 (2.39)	9.18 (3.13)	0.19
Gender: female (%)	75.0	72.2	88.2	0.48
Mean HRSD Score	11.50 (5.48)	11.94 (3.98)	12.06 (4.48)	0.94
Diagnosis by SCID (%)				0.55
MDD-PR	37.5	16.7	23.5	
MDD-MiD	18.8	38.9	23.5	
MDD-MoD	43.8	44.4	52.9	
Antidepressants prescribed (%)				0.42
SSRIs	56.3	81.3	58.8	
SNRIs	6.3	12.5	5.9	
TCAs	18.8	0	23.5	
Others^#^	18.8	6.3	11.8	
Hypnotic prescribed (%)	25.0	23.5	29.4	0.94
Sleep variables				
HRSD-sleep items	1.88 (1.46)	2.00 (1.41)	2.63 (2.09)	0.41
TST (hours/night)	6.23 (2.47)	6.68 (1.88)	6.21 (2.05)	0.69
SOL (minutes/night)	44.33 (60.08)	28.28 (20.40)	48.28 (46.25)	0.44
WASO (minutes/time)	29.00 (36.14)	29.72 (31.53)	33.65 (25.50)	0.90

*Note. *WL: waitlist control; CBT: cognitive-behavioural therapy; DMBI: *dejian* mind-body intervention; HRSD: hamilton psychiatric rating scale for depression; SCID: structural clinical interview for DSM-IV; MDD: major depressive disorder; PR: partial remission; MiD: mild depressive episode; MoD: moderate depressive episode; SSRIs: selective serotonin re-uptake inhibitors; SNRIs: serotonin-norepinephrine reuptake inhibitors; TCAs: tricyclic antidepressants; TST: total sleep time; SOL: sleep onset latency; WASO: wake time after sleep onset. Standard deviations are in parentheses. ^ #^Prescription of other types of antidepressant or a combination of SSRIs with other type of antidepressant.

**Table 2:** Psychiatrist rating on the sleep items of HRSD at pre- and post-treatment.

Group	Pre-treatment	Post-treatment	Post minus Pre Difference	*t*	*P*	Effect size
WL (*n* = 16)	1.88 (1.46)	2.81 (2.01)	0.94 (2.29)	−1.64	0.06	0.41^+^
CBT (*n* = 15)	2.00 (1.41)	1.47 (1.46)	−0.53 (2.00)	1.04	0.16	0.27
DMBI (*n* = 16)	2.63 (2.09)	1.13 (1.26)	−1.50 (1.51)	3.99	0.00**	1.00^++^

*Note.* HRSD: hamilton psychiatric rating scale for depression; WL: waitlist control; CBT: cognitive-behavioural therapy; DMBI: *Dejian* mind-body intervention. Standard deviations are in parentheses. **P < 0.001, ^+^medium effect size, ^++^large effect size.

**Table 3:** Total sleep time (hours) at pre- and post-treatment.

	Pre-treatment	Post-treatment	Post minus Pre Difference	*t*	*P*	Effect size
Whole group						
WL (*n* = 15)	6.23 (2.47)	5.57 (2.37)	0.67 (1.93)	1.34	0.10	0.35
CBT (*n* = 17)	6.68 (1.88)	6.65 (2.21)	−0.03 (1.397)	0.09	0.47	0.02
DMBI (*n* = 17)	6.21 (2.05)	7.00 (2.54)	0.79 (1.64)	−2.00	0.03*	0.48^+^
Subgroup						
WL (*n* = 7)	4.14 (1.07)	4.36 (1.49)	0.21 (1.15)	−0.49	0.32	0.19
CBT (*n* = 5)	4.60 (0.82)	4.30 (1.15)	−0.30 (1.04)	0.65	0.28	0.29
DMBI (*n* = 6)	4.08 (1.24)	5.17 (0.68)	1.08 (1.36)	−1.96	0.05*	0.80^++^

*Note.*WL: waitlist control; CBT: cognitive-behavioural therapy; DMBI: *Dejian* mind-body intervention. Subgroup included participants who totally slept for <6 hours/night at baseline. Standard deviations are in parentheses. *P ≤ 0.05, ^+^medium effect size, ^++^large effect size.

**Table 4:** Sleep onset latency (minutes) at pre- and post-treatment.

	Pre-treatment	Post-treatment	Post minus Pre Difference	*t*	*P*	Effect size
Whole group						
WL (*n* = 15)	44.33 (60.08)	57.83 (41.74)	13.50 (59.96)	−0.87	0.20	0.23
CBT (*n* = 16)	28.28 (20.40)	30.41 (23.52)	2.13 (20.73)	−0.41	0.34	0.10
DMBI (*n* = 16)	48.28 (46.25)	38.47 (38.81)	−9.81 (21.25)	1.85	0.04*	0.46^+^
Subgroup						
WL (*n* = 4)	67.50 (15.00)	90.00 (24.50)	22.50 (28.72)	−1.57	0.11	0.78^++^
CBT (*n* = 5)	53.00 (9.75)	40.00 (18.71)	−13.00 (13.04)	2.23	0.05*	1.00^++^
DMBI (*n* = 6)	97.50 (39.97)	80.00 (30.98)	−17.50 (30.62)	1.40	0.11	0.57^+^

*Note.* WL: waitlist control; CBT: cognitive-behavioural therapy; DMBI: *Dejian* mind-body intervention. Subgroup included participants who took >30 minutes to fall asleep at baseline. Standard deviations are in parentheses. *P < 0.05, ^+^medium effect size, ^++^large effect size

**Table 5:** Wake time after sleep onset (minutes) at pre- and post-treatment.

	Pre-treatment	Post-treatment	Post minus Pre Difference	*t*	*P*	Effect size
Whole group						
WL (*n* = 10)	29.00 (36.14)	28.45 (35.54)	−0.55 (33.56)	0.05	0.48	0.02
CBT (*n* = 16)	29.72 (31.53)	21.75 (19.11)	−7.97 (22.45)	1.42	0.09	0.36
DMBI (*n* = 10)	33.65 (25.50)	21.55 (19.92)	−12.10 (26.34)	1.45	0.09	0.46^+^
Sub-group						
WL (*n* = 5)	49.50 (42.88)	44.00 (44.64)	−5.50 (48.62)	0.25	0.41	0.11
CBT (*n* = 9)	51.11 (26.19)	30.56 (19.44)	−20.56 (20.68)	2.98	0.01*	0.99^++^
DMBI (*n* = 6)	51.67 (13.29)	28.83 (22.23)	−22.83 (28.53)	1.96	0.05*	0.80^++^

*Note.* WL: waitlist control; CBT: cognitive-behavioural therapy; DMBI: *Dejian* mind-body intervention. Subgroup included participants who took >15 minutes to resume sleep after awakening at baseline. Standard deviations are in parentheses. *P ≤ 0.05, ^+^medium effect size, ^++^large effect size.
